# Soil Carbon Dioxide Emissions and Carbon Sequestration with Implementation of Alley Cropping in a Mediterranean Citrus Orchard

**DOI:** 10.3390/plants13172399

**Published:** 2024-08-28

**Authors:** Jose A. Acosta, Alberto Imbernón-Mulero, Belén Gallego-Elvira, Jose F. Maestre-Valero, Silvia Martínez-Martínez, Victoriano Martínez-Álvarez

**Affiliations:** Department of Agricultural Engineering, Technical University of Cartagena, Paseo Alfonso XIII 48, 30203 Cartagena, Spain

**Keywords:** *Thymus hyemalis*, *Rosmarinus officinalis*, grapefruit, intercropping, greenhouse gas emissions, climate change

## Abstract

Agroecological ecosystems produce significant carbon dioxide fluxes; however, the equilibrium of their carbon sequestration, as well as emission rates, faces considerable uncertainties. Therefore, sustainable cropping practices represent a unique opportunity for carbon sequestration, compensating greenhouse gas emissions. In this research, we evaluated the short-term effect of different management practices in alleys (tillage, no tillage, alley cropping with *Rosmarinus officinalis* and *Thymus hyemalis* on soil properties, carbon sequestration, and CO_2_ emissions in a grapefruit orchard under semiarid climate). For two years every four months, soil sampling campaigns were performed, soil CO_2_ emissions were measured, and rhizosphere soils were sampled at the end of the experimental period. The results show that alley cropping with *Thymus* and *Rosmarinus* contributed to improve soil fertility, increasing soil organic carbon (SOC), total nitrogen, cation exchange capacity, and nutrients. The CO_2_ emission rates followed the soil temperature/moisture pattern. Tillage did not contribute to higher overall CO_2_ emissions, and there were no decreased SOC contents. In contrast, alley crops increased CO_2_ emission rates, especially *Rosmarinus*; however, the bigger root system and biomass of *Rosmarinus* contributed to soil carbon sequestration at a greater rate than *Thymus*. Therefore, *Rosmarinus* is positioned as a better option than *Thymus* to be used as an alley crop, although long-term monitoring is required to evaluate if the reported short-term benefits are maintained over time.

## 1. Introduction

Greenhouse gas (GHG) emissions constitute a critical contemporary challenge, profoundly impacting our environment and the global climate [1,2]. Carbon dioxide (CO₂) emissions account for over 75% of global GHG emissions and nearly 90% of all anthropogenic CO₂ emissions [3]. Particularly, GHG emissions from agricultural resources contribute 11–30% to global anthropogenic GHG emissions [4,5]. Agricultural GHG emissions primarily result from specific management practices. These include soil management activities such as tillage, the utilization of both inorganic and organic fertilizers, livestock management, the use of fossil fuels, and the combustion of agricultural residues during land use changes [6,7]. As a consequence, agroecological ecosystems produce significant CO₂ fluxes; however, the equilibrium of their carbon sequestration, as well as emission capabilities, faces considerable uncertainties, as well as high annual and spatial variability [8,9]. Therefore, sustainable cropping practices represent a unique opportunity for carbon sequestration, compensating GHG emissions [10].

In this context, soils play a critical role in the global carbon cycle and are essential for mitigating climate change [11]. They act as both carbon sinks, storing significant amounts of terrestrial carbon, and as regulators of GHG emissions. When managed sustainably, soils contribute to carbon sequestration, helping to reduce atmospheric CO₂ levels and enhance resilience against climate change. Globally, soils are the biggest terrestrial carbon sink, storing more than twice as much carbon than the vegetation and atmosphere [12,13]. This means that as global temperatures rise due to global warming, soil respiration may increase, leading to higher CO₂ emissions. These emissions could potentially contribute to a positive feedback loop, exacerbating climate warming [14,15].

Soil respiration involves several interconnected processes, including the rhizosphere priming effect (RPE), as well as rhizodeposition and litter decomposition. In the RPE, the presence of living plant roots stimulates the decomposition of soil organic matter. Essentially, the roots release compounds into the soil, providing energy sources for soil microorganisms. As a result, the microbial activity increases, leading to enhanced soil respiration and the release of CO₂ into the atmosphere, as well as rhizodeposition and litter decomposition. Rhizodeposition involves the direct release of organic compounds by roots into the soil, which serve as substrates for microbial growth and metabolism, influencing soil respiration rates. Litter decomposition refers to when plant material (such as fallen leaves or other organic debris) accumulates on the soil surface: it undergoes decomposition, and then soil microorganisms break down this litter, releasing CO₂ during the process [16,17].

Numerous studies have investigated how agricultural practices contribute to the rise in atmospheric greenhouse gas concentrations, especially carbon dioxide [18,19]. The literature on soil carbon dynamics and climate change presents conflicting findings regarding the ability of agricultural practices to enhance carbon sequestration in soils while reducing greenhouse gas emissions [20]. However, it has been demonstrated that intensive agricultural systems result in higher greenhouse gas emissions and a reduction in soil organic matter (SOM) due to low residue turnover, lack of year-round soil cover, and frequent tillage, which accelerates SOM mineralization [21,22,23]. In Mediterranean tree orchards, the situation is exacerbated. Trees are irrigated, and the alleys are intentionally kept bare to prevent competition for water and nutrients with weeds or other crops. Furthermore, a longstanding social perception associates maintaining vegetation cover in the alleys with neglect and dirt, leading to weed control through tillage or herbicide application [24,25]. Consequently, soil erosion rates in Mediterranean orchards are high, having significant implications for soil health and sustainability, such as the high mineralization of SOM and GHG emissions and poor structure and low content of SOM and nutrients, leading to a low capacity to hold water and favor infiltration [26,27].

In addition, in the context of climate change, rising temperatures have a significant impact on soil organic carbon (SOC) storage, since as temperatures increase, the decomposition rate of the SOM accelerates, and the faster decomposition leads to a release of CO₂ into the atmosphere, reducing the overall storage of SOC [28]. This phenomenon is exacerbated by drier growing seasons, as shown by Ko-chiieru et al. [29], because CO_2_ is generated in the soil through biochemical reactions linked to the biological activity of microorganisms and root respiration, which are primarily influenced by soil temperature and moisture [30,31]. Therefore, sustainable croplands management has a high potential to mitigate climate change, but this requires a deep understanding of soil carbon dynamics and how agricultural management, and environmental variables affect them, being necessary to promote sustainable agricultural management practices that contribute to SOC sequestration while reducing GHG emissions [32].

To maintain existing SOC stocks and restore SOC in carbon-depleted soils, several sustainable agricultural practices are recommended: reducing tillage intensity and frequency, growing cover crops in alleys, the application of compost or manure, leaving crop residues on the fields after harvest, etc. [33,34,35,36]. In this way, crop diversification remains a promising strategy with significant potential for further development to address these challenges, particularly intercropping system, using perennial vegetation cover in the alleys, which has been related to increased agrosystem resilience [37]. Intercropping is an agricultural practice where two or more crops are grown together in the same field during the same growing season. This method aims to maximize the use of resources such as sunlight, water, and nutrients, leading to higher overall productivity compared to growing a single crop. Intercropping offers several benefits, such as improved soil fertility, better pest and disease control, and the more efficient use of resources. In arid regions, crop diversification serves as a promising approach to combat climate change and soil degradation by sequestering carbon in the soil while safeguarding water and biodiversity [38,39,40]. In addition, alley cropping contributes to decreased GHG emissions, increased productivity, improved soil quality, and water holding capacity, and it attracts pollinators and auxiliary fauna [41,42]. In fact, this strategy is aligned with the European Green Deal [43] and the European Climate Law [44], which aim to make a fair transition in the EU’s economy to achieve climate neutral farms by 2050 [45].

It should be considered that the plants used in the alleys crop must be species adapted to the soil and climatic conditions of the area, with the aim of allowing them to develop optimally. Additionally, they should be compatible with the machinery and agriculture practices of the main crop [46]. In this regard, the use of aromatic species (e.g., *Thymus, Lavandula, Rosmarinus, Salvia*, *Helichrysum*, *Artemisia*, etc.) is a promising option, as it requires minimal maintenance by the farmer and could obtain economic incomes in addition to environmental ones, such as selling them to the cosmetics industry for their essential oils or as ingredients for the food industry [47,48].

The main aim of this study was to assess the effect of a two-year implementation of different management practices in alleys, namely, tillage, no tillage, and alley cropping with *Rosmarinus* and *Thymus*, on the soil physicochemical properties, carbon sequestration, and CO_2_ emissions in a citrus orchard (grapefruit) under semiarid Mediterranean conditions.

## 2. Results

### 2.1. Physicochemical Characteristics of Soils

Table 1 shows the physicochemical characteristics of the soils before and 2 years from the beginning of the treatments. Since the initial alley soil conditions correspond to tillage for a time, it would be expected that the results of the tillage treatment would hardly change the initial physicochemical characteristics after two additional years of tillage. However, tillage caused a statistically significant decrease in pH (8.5 to 8.2) and water-soluble chloride concentration (41.9 to 12.4 mg L^−1^), as well as an increase in CEC (from 10.7 to 13.8 cmol kg^−1^) and available Ca concentrations (from 1634 to 2177 mg kg^−1^).

The experimental data results indicate that soil pH from no-tillage alley also decreased from 8.5 to 8.2, and the concentrations of water-soluble chlorides (from 41.9 to 17.4 mg L^−1^) and sulfates (from 43.5 to 15.7 mg L^−1^) changed, increasing waster-soluble nitrate concentrations (from 18.6 to 55.4 mg L^−1^). In addition, when no-tillage and tillage alleys are compared, after two years of treatment, only a decrease in the content of water-soluble nitrates was observed after tillage (from 55.4 to 16 mg L^−1^).

The results show that the presence of Rosmarinus and Thymus in the alleys reduced the soil pH, especially in the rhizosphere soils—from 8.5 to 7.3. In contrast, in the rhizosphere of both plants, an increase in electrical conductivity (EC); organic carbon (OC); total nitrogen (TN); cation exchange capacity (CEC); available Na, Ca, and Mg; and water-soluble Na were observed with respect to the initial characteristics of the alley soils (Table 1). Furthermore, in the Thymus rhizosphere, the concentrations of available Fe and Mn, as well as water-soluble Mg and sulfates, also were increased, while the concentration of water-soluble nitrates decreased compared to the levels found in the alley.

Similarly, the bulk soils of both species (Thymus and Rosmarinus) presented higher OC contents, as well as available and water-soluble Na, than those initially found in the alleys (Table 1). Also, in the bulk soils of Thymus, an increase in EC was observed, and decreases in water-soluble chlorides and sulfates were recorded, while in the bulk soils of Rosmarinus, increases were also observed in the CEC; available Mg; and in water-soluble K, Ca, Mg, and sulfates concentrations, as well as a decrease in water-soluble nitrate concentrations when compared to the alley.

When the effect of the rhizosphere is compared with the bulk soil of both species (*Rosmarinus* and *Thymus*) (Table 1), it can be observed that the rhizosphere of *Rosmarinus* reduced the pH of the soils (from 8.2 to 7.3), as well as the concentration of K (from 24.6 to 11.6 mg L^−1^), water-soluble Ca (from 59.9 to 23.4 mg L^−1^), Mg (from 16.8 to 4.2 mg L^−1^), and sulfates (from 66 to 37.6 mg L^−1^), while the EC (from 0.3 to 0.7 dS m^−1^), OC (from 1.2 to 1.4 %), TN (from 0.14 to 0.24 %), CEC (from 14 to 18.9 cmol kg^−1^), available Na (from 136 to 390 mg kg^−1^), and Mg (from 300 to 459 mg kg^−1^) contents, as well as the water-soluble nitrates (from 4.1 to 15.7 mg L^−1^) were increased. On the other hand, the *Thymus* rhizosphere reduced the soil pH (from 8.4 to 7.3) while increasing TN (from 0.12 to 0.16%), CEC (from 10.8 to 18.2 cmol kg^−1^), available Fe (from 8.7 to 13.9 mg kg^−1^), Mn (from 7.4 to 12.4 mg kg^−1^), Na (from 153 to 267 mg kg^−1^), and Ca (from 1846 to 2812 mg kg^−1^) contents, as well as water-soluble Na (from 37.8 to 48.1 mg L^−1^), Mg (from 4.4 to 10.9 mg L^−1^), chlorides (from 9.8 to 33.5 mg L^−1^), and sulfates (from 6.6 to 91.8 mg L^−1^).

Comparing both alley croppings, the bulk soils of *Rosmarinus* presented higher concentrations of water-soluble K, Ca, Mg, sulfates, and chlorides than those reported for the bulk soils of *Thymus*. In addition, the EC, OC, TN, available Mg, and water-soluble nitrates were higher in the rhizospheric soils of *Rosmarinus* than the rhizospheric soils of *Thymus*. In contrast, available Fe, Mn, Mg, and water-soluble Na, Mg, and nitrates were higher in the rhizospheric soils of *Thymus*.

Finally, after two years, the grapefruit soils decreased their salinity, as well as the concentrations of available Na, water-soluble Na, K, Ca, Mg, chlorides, sulfates, and nitrates, increasing their OC content.

### 2.2. Carbon Dioxide Emission Rates

**Figure 1:**
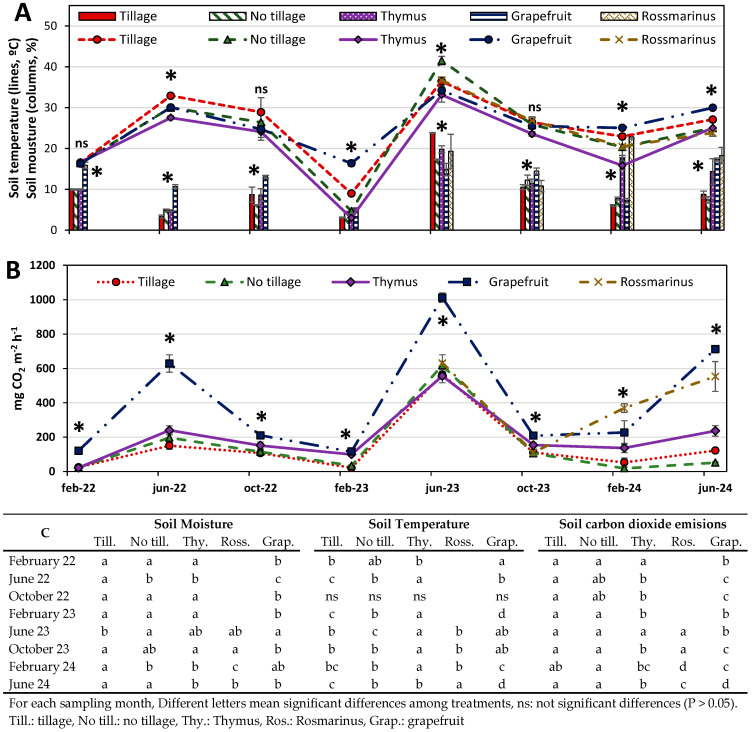
Soil temperature and soil moisture during the experiment (**A**), soil CO_2_ emission rates (**B**), and statistically significant differences (**C**) from the different treatments. Different letters and * mean significant differences among treatments; ns: no significant differences (*p* > 0.05).

The soil temperature varied seasonally (Figure 1A), with the highest values recorded in the months of June, with a maximum in June-23 (41–33 °C) and the lowest in the months of February, with the minimum value in February-23 (3–16 °C), with significant differences between treatments. The soil moisture also presented statistically significant differences between treatments (Figure 1A), whose variations were due to the irrigation carried out on the grapefruits, as well as the rainfalls during the experimental period, being in all cases below 25% of volumetric humidity.

The soil CO_2_ emission rates followed a similar trend to both soil temperature and moisture, with a positive correlation with both parameters (with soil temperature R = 0.636, *p* > 0.05, and with soil moisture R = 0.631, *p* > 0.05). The highest CO_2_ emission rates were measured when the soil temperature and moisture were highest, coinciding with the summer month (Figure 1B).

The carbon dioxide emissions were statistically higher in the grapefruit crop than in the rest of the treatments throughout all of the experimental period, with a mean value of 405 mg CO_2_ m^−2^ h^−1^, except for February-23, where it was similar to the CO_2_ emissions in the *Thymus* alley (116–99 mg CO_2_ m^−2^ h^−1^); in February-24, they were lower than the CO_2_ emissions in the *Rosmarinus* alley (227–370 mg CO_2_ m^−2^ h^−1^) (Figure 1B,C).

No statistically significant differences were observed in the CO_2_ emissions in the tillage and no-tillage alleys in any of the measurements carried out, with mean values for the entire experimental period of 144 and 145 mg CO_2_ m^−2^ h^−1^, respectively (Figure 1B,C). The CO_2_ emission rates in the *Rosmarinus* alley were statistically higher than the emissions from the *Thymus* alley, except for June-23, where they were similar (631–556 mg CO_2_ m^−2^ h^−1^, respectively), and October-23, where they were lower (112–154 mg CO_2_ m^−2^ h^−1^, respectively), with a mean value for the entire experimental period of 416 and 200 mg CO_2_ m^−2^ h^−1^, respectively. We highlight that those soils planted with *Thymus* had higher CO_2_ emissions rates than the tillage soils in most of the measurements carried out, with mean values of 200 and 144 mg CO_2_ m^−2^ h^−1^, respectively.

### 2.3. Soil Inorganic/Organic Carbon and Total Nitrogen Evolution in Soils

**Figure 2:**
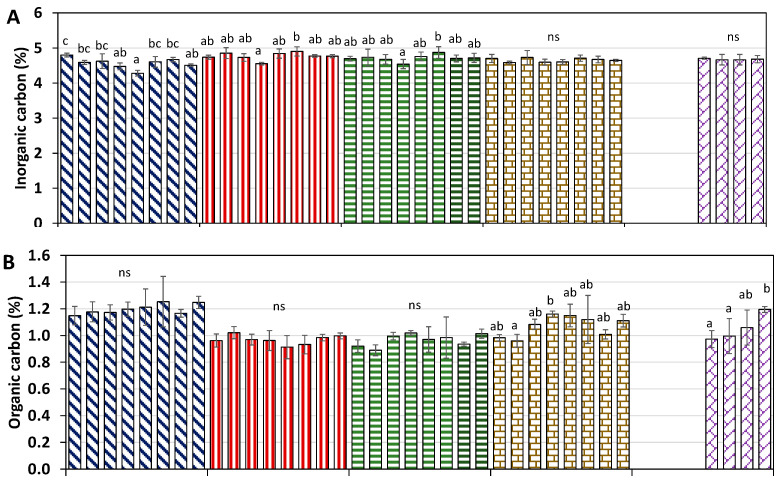

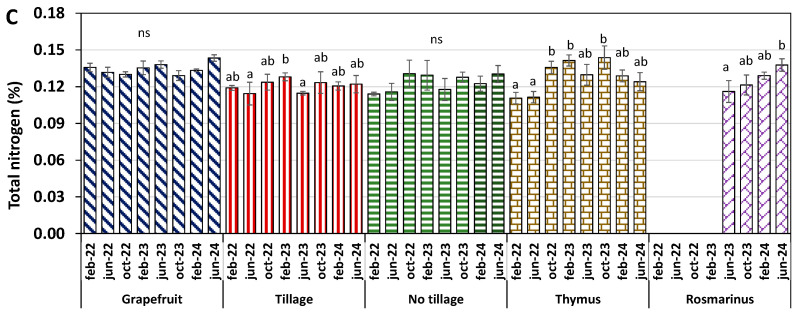
Seasonal changes of inorganic carbon (**A**), organic carbon (**B**), and total nitrogen (**C**) in the orchard soil under different treatments (no tilling, tilling, *Rosmarinus officinalis*, *Thymus hyemalis*, and grapefruit). Error bars denote standard deviation. Different letters mean significant differences among treatments; ns: not significant differences (*p* > 0.05).

The soil inorganic carbon (IC) content was close to 5% in all treatments and throughout the study period (Figure 2A), with no statistically significant differences in the alleys planted with *Thymus* or *Rosmarinus*. However, differences were observed in the rest of the treatments (grapefruit, tillage, and no tillage), although in no case were the values below 4%.

The soil OC contents were higher in the soils with grapefruit than in the rest of the treatments, with percentages close to 1.2%, while in the tillage and no-tillage alleys, the OC values were close to 1%, with no significant differences neither between these two treatments nor throughout the experimental period (Figure 2B). On the contrary, in the alleys planted with *Thymus* and *Rosmarinus*, statistically significant differences were observed over time. However, in the case of *Thymus*, the differences were only observed between June-22 and February-23, with no trend of increase over time, returning to initial OC values 2 years after planting. In contrast, in the *Rosmarinus* alley, a significant increase in OC content was observed one year after its plantation, increasing from 1% to 1.2%.

Finally, the TN content followed a similar trend as the OC content (Figure 2C), with the highest values in the grapefruit soils (0.14%) compared to the rest of the treatments (0.12%). No significant differences were found throughout the experimental period for grapefruit and no-tillage alleys, keeping the TN levels constant over time. Contrarily, significant differences were reported in tillage soils and those planted with *Thymus* and *Rosmarinus*. However, in the tillage and *Thymus* alleys, these differences were not increasing over time, presenting similar values to the initial ones after two years of treatment. In contrast, in the *Rosmarinus* alley, a significant increase was observed one year after planting, which is correlated with the soil OC content (R = 0.771, *p* > 0.05).

## 3. Discussion

### 3.1. Effect of Alley Cropping on Physicochemical Characteristics of Soils

The pHs of the study area are slightly alkaline [49] due to the high presence of carbonates in the soils [50], where a decrease occurred during the experimental period in both the tillage and no-tillage treatments (Table 1), probably because of soil turning during tillage and changes in the cations bound to exchange complex [51]. The rainfalls that occurred during the study period caused the leaching of chlorides and sulfates in the no-tillage alley, as well as nitrates in the tillage alley, which are easily mobilizable elements in the soil profile [52].

Although tillage is known to decrease soil OC and TN with negative consequences for soil fertility [53], no differences were found for the OC and TN contents after tillage. Therefore, two years is not enough time to see the effects of tillage on these soil constituents. Furthermore, the study orchard has been tilled for a long period of time, approximately 12 years, reaching a state of equilibrium, which cannot be modified with only two years of no tillage. However, other studies concluded that no-tillage soils had higher soil OC concentration at the topsoil (0–10 cm) as compared to tillage soils, because tillage inverts the soil surface and distributes soil OC in the deep soil profile [54], which could also explain the increase in CEC observed in the tillage soils (Table 1), where finer soil particles (clay and silt) were placed on the soil surface after tillage.

The alley crops improved the soil fertility, especially for rhizospheric soils, since they increased the contents of OC, TN, CEC, as well as the concentration of nutrients (Ca and Mg) (Table 1). It is known that root release exudates organic carbon compounds, such as simple sugars, organic acids, and amino acids, that promote the nutrient release [55]; likewise, plant debris are incorporated into the soil, increasing the contents of OC and TN [56]. The increase in soil OC has a positive effect on the CEC of the soil and the ability to retain nutrients and to remain fertile [57]. In addition, root exudates of the alley crops may have stimulated and activated soil microbial communities [58,59], contributing to the humidification process and incorporation of organic matter into the soil.

Comparing bulk soils of both alley cropping, the presence of *Rosmarinus* increased the salinity of the soil due to a greater presence of soluble anions/cations, which could be because this species had a bigger and well-developed root system, with a greater exudation of acidic organic substances, such as citrate, malate, and oxalate, which promoted nutrient mobilization, increasing their soluble concentrations [60]. Similarly, the greater biomass of *Rosmarinus* caused the concentrations of OC and TN to be higher in its rhizosphere than in the *Thymus* rhizosphere, which were 1.4 and 1.2% of OC and 0.24 and 0.16% of TN, respectively.

### 3.2. Evolution of Carbon Dioxide Emissions

Soil CO_2_ production is a biochemical process linked to biological activities like root respiration and the decomposition of organic matter by microbial activity, which are influenced by soil temperature, moisture, and nutritional status [61,62]. In the present study, there were significant seasonal variations in the soil CO_2_ efflux of all treatments. The results indicate that soil CO_2_ fluxes were low during the winter period (February) and increased during the summer period (June) (Figure 1A,B), coinciding with periods of higher temperature and soil moisture [63]. We observed significant positive correlations of soil temperature and soil moisture with CO_2_ emission rates at the same intensity, providing evidence that CO_2_ fluxes may be controlled by the soil environmental condition [64,65]. In fact, among environmental factors, the temperature and moisture content are considered to have the greatest control on soil CO_2_ fluxes [66,67]. However, Zornoza et al. [23] reported the greater dominance of temperature on GHG emission control in semiarid orchards. Other experimental studies have consistently observed that CO_2_ fluxes are highest at moderate soil moisture levels [68], while at low soil moisture, CO_2_ fluxes are restricted due to the limited diffusion of organic carbon substrates and physiological stress on cells [69].

Even though no tillage is an alternative to mitigate GHG emissions from agricultural practices [6,35,70], the results from our study showed no statistically significant differences between tillage and no-tillage alleys, which could be due to the low organic carbon content of the soils (Figure 2B) and the low microbiological activity in soils from arid and semiarid climates [71]. Moitinho et al. [18] found that soil CO_2_ emissions were strongly influenced by soil microbial dynamics and showed a positive correlation with microbial biomass carbon.

The use of alley crops increased the CO_2_ emission rates when compared with tillage alleys, especially when the alley crops were well developed (Figure 2B,C). Authors such as [72] found that the presence of a crop could promote higher CO_2_ production. Nine months after planting *Rosmarinus*, it was observed that the CO_2_ emission rates were higher for *Rosmarinus* than for *Thymus*, which is likely due to the greater root development of this species and greater microbial activity in its rhizosphere [73,74].

### 3.3. Carbon Sequestration in Soils

Intensive agriculture depends on robust and fertile soils to maintain sustainable and stable yields, while also reducing environmental impacts, especially in the face of climate change [75]. In order to increase soil fertility, it is necessary increase soil OC through sustainable agricultural practices, since enhancing soil OC offers significant potential for boosting soil fertility. In addition, soil OC influences numerous soil properties, including physical, chemical, and biological aspects, such as water retention, nutrient cycling, and microbial activity [76].

One of the most implemented agricultural practices for increasing soil OC is the application of no-tillage systems. No-till farming is regarded as a low-carbon agricultural practice that enhances soil carbon stocks within a few years of adoption [77,78,79]. However, results from the mentioned study indicated that two years after its implementation, no differences were observed in the tillage alleys compared to the no tillage ones, presenting the same soil OC content (Figure 2B), which is due to the many years of tillage of the soils of the alleys studied (~12 years); therefore, a longer time is needed to observe the positive benefices of these systems under the semiarid environmental conditions of the study area.

In contrast, in the present study, alley crops increased the content of soil OC; however, this increment was only sustained over time in *Rosmarinus* alley, likely due to a its faster and vigorous development, where its root and leaf debris represent direct carbon inputs to the soil, especially in the soil surface, which can be decomposed and transformed into a stable source of organic matter [79]. Although the soil OC concentration was less than 1.2% at the end of the experimental period, the values increased by 20% after 2 years of cultivation compared to the initial value (Figure 2B), which indicated increased OC sequestration under the current conditions. However, Ben Moussa-Machraoui et al. [80] did not observe a significant increase in soil organic carbon (SOC) in their study following the implementation of no tillage with intercrop diversification. They attributed this to the short duration of minimum tillage use or alterations in soil structure due to prolonged conventional tillage. Therefore, long-term monitoring is recommended to evaluate if the observed increase in soil OC under *Rosmarinus* is continuing over time.

## 4. Materials and Methods

### 4.1. Study Area and Soil Sampling

**Figure 3:**
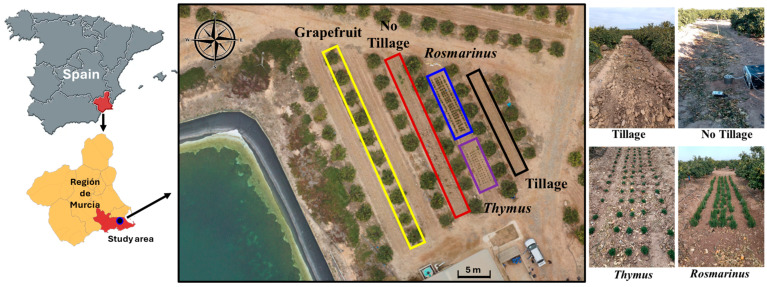
Location of the study area and experimental design.

The experimental work was carried out at an open-air commercial farm located in Torre Pacheco, Region de Murcia, Spain (37°47′30″ N; 1°03′85″ W; 30 m above sea level), between February 2022 and June 2024 (Figure 3). The area is characterized by a Mediterranean semiarid climate with overall warm, dry summers and mild winters. The average annual reference evapotranspiration (ET0) and rainfall are 1322 mm and 295 mm, respectively. According to the Word Reference Base for Soil Resources [81], the soils of the study area have been classified as Haplic Calcisols (Aric). The orchard consisted of 0.28 ha cultivated with ‘Rio Red’ grapefruit trees (*Citrus* × *paradisi* Mac.) grafted on macrophylla rootstock (*Citrus macrophylla*) that were five years old at the beginning of the experiment and had a tree spacing configuration of 5.5 m × 3.5 m.

Four different treatments were stablished in the alleys of the main crop (grapefruit): (i) conventional tillage in all alley surfaces (plow 2 times yr^−1^ at 20 cm depth); (ii) no tillage in the alley; (iii) diversified with the aromatic species *Thymus hyemalis* as alley crop; and iv) diversified with the aromatic species *Rosmarinus officinalis* as alley crop (Figure 3). These two species were selected as alley cropping because they are native to the area, spontaneously growing in the surroundings, and have commercial interest, since the sale of essential oil can diversify crop production in the farm and provide extra income for the farmer [45]. It should be noted that alleys in the orchard were subjected to conventional tillage for 12 years prior to the trial. No pesticides were applied during the experiment duration. Diversified alleys were occasionally irrigated to ensure proper establishment of *Rosmarinus* and *Thymus*.

Every four months, soil sampling campaigns were performed: February-22, June-22, October-22, February-23, June-23, October-23, February-24, June-24—collecting bulk soil samples at 0–20 cm depths with an auger, since the improvements regarding soil organic carbon in agroforestry systems have concentrated on changes in this topsoil layer [82]. Three composite samples derived from 3 random subsamples were collected in each treatment (tillage, no tillage, *Rosmarinus, Thymus*); also, same sampling procedure was done for the main crop (grapefruit). In addition, three replicates of rhizospheric soils from *Rosmarinus* and *Thymus* were sampled at the end of the experimental period (June-24).

### 4.2. Analytical Methods

Soil was air-dried for one week and sieved at <2 mm. Soil pH and electrical conductivity (EC) were measured in deionized water (1:2.5 and 1:5 *w*/*v*, respectively). The percentages of sand, clay, and silt were determined using the Bouyoucos Hydrometer Method [83]. Total organic carbon (OC), total inorganic C (IC), and total nitrogen (TN) were determined using LECO CHNS Elemental Analysis. Water soluble cations (Ca, Mg, Na, K) and anions (sulphates, chlorides, and nitrates) were extracted with deionized water in a 1:5 soil:extractant ratio and measured using ion chromatography for anions (Dionex DX 500, Waltham, MA, USA) and inductively coupled plasma mass spectrometer for cations (ICP-MS, Agilent Technologies, Model 7900, Santa Clara, CA, USA) [84]. Cation exchange capacity (CEC) was determined according to Soil Survey Staff [85] and available cations (Ca, Na, K, Mg) measured with an inductively coupled plasma mass spectrometer (ICP-MS, Agilent Technologies, Model 7900, Santa Clara, CA, USA). Available phosphorus (P) was measured using the sodium bicarbonate extraction-based Olsen P method [85], while the bioavailable minor elements (Zn, Fe, Cu, and Mn) were estimated using the diethylene-triamine pentaacetic acid (DTPA) method [86] with an inductively coupled plasma mass spectrometer (ICP-MS, Agilent Technologies, Model 7900, Santa Clara, CA, USA).

### 4.3. Soil Carbon Dioxide Measurements

Measurements of soil CO_2_ were seasonally made every 4 months in all replicated treatments from 5 February 2022 to 5 July 2024 between 9:00 and 11:00; at the same times and points, soil moisture and temperature were also recorded with a portable probe (Procheck C, Decagon 5TM, METER Group, München, Germany). These measurements were taken simultaneously with the soil sampling campaigns. The primary experimental procedure employed in this study was the dynamic gas chamber technique. The chamber, made of non-oxidizable steel, had a diameter of 7.5 cm and a height of 20 cm, with one inlet and one outlet connected to a photoacoustic infrared spectroscopy multi-gas analyzer featuring an ultra-sensitive cantilever pressure sensor (GASERA One, Gasera Ltd., Turku, Finland). This dynamic system, with both an inlet and outlet, allows for continuous flow and prevents pressure fluctuations. The chambers were placed on a non-oxidizable steel base, which was randomly inserted into the bare soil to a depth of 15 cm in the middle of the alleys. Chambers were randomly inserted between two plants. CO_2_ amounts were quantified every 1 min for a period of 5 min to assess the linear trend. CO_2_ emissions rates were expressed in mg m^−2^ h^−1^ as the difference between the quantification at the end and the beginning of the measure period divided by the time.

### 4.4. Statistical Analysis

Data were verified for normal distribution using the Kolmogorov–Smirnov test at *p* < 0.05. Homoscedasticity was assessed with Levene’s test, and data were log-transformed when necessary. For comparing the effect of different treatments (tillage, no tillage, *Rosmarinus*, *Thymus*, and grapefruit) on soil physical–chemical properties (before and after treatments), soil data were submitted to one-way ANOVA, and Tukey’s Honestly Significant Difference (HSD) test at *p* < 0.05. Carbon dioxide emissions were also submitted, independently for each measure date, to one-way ANOVA and Tukey’s Honestly Significant Difference (HSD) test at *p* < 0.05 to compare significant differences between treatments. Finally, inorganic/organic carbon and total nitrogen measures every four months were also submitted to one-way ANOVA and Tukey’s Honestly Significant Difference (HSD) test at *p* < 0.05 to evaluate the evolution of these soil constituents and compare significant differences between sampling periods. Spearman correlation test was used to examine the relationships between soil inorganic/organic carbon and total nitrogen and CO_2_ emission rates.

## 5. Conclusions

Alley cropping with *Thymus* and *Rosmarinus* contributed to improve soil fertility in alleys of a grapefruit orchard from the Mediterranean climate, whose effects were observed with greater intensity in the rhizospheric soils of both species, since the content of soil OC, TN, CEC, and concentration of nutrients increased.

The results showed that the soil CO_2_ emission rates were regulated by soil temperature and moisture, with higher CO_2_ emissions when soil temperature and moisture were higher. No differences were observed for the CO_2_ emission rates when the no-tillage system was implemented, accordingly, tillage did not contribute to higher overall CO_2_ emissions, likely due to low content of soil OC and the low microbiological activity in the alleys. In addition, no tillage did not contribute to increased OC content in the alley soils, since no differences between the tillage and no-tillage alleys were found.

In contrast, alley crops increased the CO_2_ emission rates when compared with tillage and no-tillage alleys, especially for *Rosmarinus*, whose CO_2_ emission rates were higher than those reported for *Thymus*. However, although both species contributed to soil carbon sequestration, *Rosmarinus* increased the soil OC content to a greater extent than *Thymus*, and since the aerial biomass of *Rosmarinus* was bigger than that of *Thymus*, it had a higher uptake of atmospheric carbon dioxide.

Therefore, according to the obtained results, *Rosmarinus* is positioned as a better option than *Thymus* for alley cropping; however, long-term monitoring is required to evaluate if the observed benefits with the implementation of alley crop are maintained over time.

## Figures and Tables

**Table 1:** Physicochemical characteristics of soils before and 2 years after treatments.

	Before Treatments		Soil 2 Years after Treatments						
	Grapefruit	Alley	Grapefruit	No tillage	Tillage	*Rosmarinus officinalis*		*Thymus hyemalis*	
						Bulk Soil	Rhiz. Soil	Bulk Soil	Rhiz. Soil
pH	7.8 ± 0.2 b	8.5 ± 0 c	8.3 ± 0 bc	8.2 ± 0.1 b	8.2 ± 0 b	8.2 ± 0 b	7.3 ± 0.1 a	8.4 ± 0 bc	7.3 ± 0.1 a
EC (dS/m)	0.8 ± 0.1 c	0.2 ± 0 a	0.2 ± 0 a	0.2 ± 0 a	0.2 ± 0 a	0.3 ± 0 b	0.7 ± 0.1 c	0.4 ± 0 b	0.5 ± 0.1 b
Inorganic carbon (%)	4.8 ± 0.1 b	4.7 ± 0.1 ab	4.5 ± 0 a	4.7 ± 0 ab	4.8 ± 0 b	4.7 ± 0.1 ab	4.9 ± 0 b	4.6 ± 0.1 ab	4.9 ± 0 b
Organic carbon (%)	1.1 ± 0.02 b	0.9 ± 0.05 a	1.2 ± 0.05 c	1 ± 0.02 a	1 ± 0.05 a	1.2 ± 0.02 bc	1.4 ± 0.04 d	1.1 ± 0.03 b	1.2 ± 0.01 bc
Total Nitrogen (%)	0.14 ± 0 abc	0.11 ± 0 a	0.14 ± 0.01 bc	0.13 ± 0.01 ab	0.12 ± 0 ab	0.14 ± 0.01 abc	0.24 ± 0.02 d	0.12 ± 0.01 ab	0.16 ± 0.01 c
CEC (cmol kg^−1^)	11.1 ± 0.5 ab	10.7 ± 0.4 a	12.8 ± 0.2 b	12.6 ± 0.4 ab	13.8 ± 0.5 b	14 ± 0.5 b	18.9 ± 0.9 c	10.8 ± 0.9 a	18.2 ± 0.9 c
Clay (%)	20.7 ± 0.9	21.2 ± 1.4	19.8 ± 0.8	21.9 ± 3.2	22.6 ± 1.1	22.6 ± 2.9	21.8 ± 3	22 ± 2.4	21 ± 1.3
Silt (%)	18.3 ± 0.7 ab	24.6 ± 1.7 c	16.4 ± 1.4 a	22.9 ± 2.6 bc	21.7 ± 2.1 bc	21.2 ± 1.4 bc	20.4 ± 0.9 abc	20.2 ± 1.7 abc	18.4 ± 1.1 ab
Sand (%)	61.1 ± 0.4 bc	54.1 ± 2.9 a	63.8 ± 1.3 c	55.1 ± 1.7 ab	55.6 ± 3.1 ab	56.2 ± 1.8 ab	57.8 ± 3.7 abc	57.8 ± 0.8 abc	60.6 ± 1.6 bc
Available P (mg kg^−1^)	96.9 ± 7.8 c	49.6 ± 3.6 ab	78.7 ± 3 c	47.3 ± 6.1 ab	39.6 ± 3.6 a	51.3 ± 7.5 ab	62 ± 7 b	48.5 ± 3 ab	57.8 ± 3.3 b
Available Fe (mg kg^−1^)	3.82 ± 0.5 a	7.61 ± 0.9 ab	5.37 ± 1 a	7.15 ± 0.7 ab	7.41 ± 1.1 ab	7.76 ± 1 ab	9.41 ± 1 b	8.7 ± 1 b	13.98 ± 1.5 c
Available Mn (mg kg^−1^)	8.63 ± 0.9 ab	8.69 ± 1 ab	8.77 ± 1 ab	8.13 ± 1.6 ab	10.91 ± 1.3 bc	8.66 ± 1.1 ab	8.87 ± 0.9 ab	7.41 ± 1.1 a	12.43 ± 1.5 c
Available Cu (mg kg^−1^)	2.07 ± 0.5	1.87 ± 0.8	1.72 ± 0.8	2.42 ± 0.6	1.79 ± 0.3	2.86 ± 0.8	2.22 ± 1.3	2.63 ± 0.8	2.63 ± 0.9
Available Zn (mg kg^−1^)	2.76 ± 0.8 ab	2.92 ± 0.6 ab	1.65 ± 0.9 a	2.63 ± 0.9 ab	2.73 ± 0.9 ab	2.82 ± 0.4 ab	2.75 ± 0.8 ab	4.38 ± 0.9 b	2.86 ± 0.8 ab
Available Na (mg kg^−1^)	142 ± 14 b	40 ± 5 a	53 ± 6 a	46 ± 4 a	52 ± 4 a	136 ± 12 b	390 ± 40 c	153 ± 26 b	267 ± 30 c
Available K (mg kg^−1^)	217 ± 25 a	247 ± 29 ab	235 ± 27 ab	246 ± 27 ab	263 ± 33 ab	296 ± 15 b	292 ± 31 ab	267 ± 32 ab	249 ± 22 ab
Available Ca (mg kg^−1^)	1650 ± 131 a	1634 ± 107 a	1613 ± 165 a	1958 ± 126 ab	2177 ± 170 b	2046 ± 58 ab	2534 ± 176 bc	1846 ± 97 ab	2812 ± 185 c
Available Mg (mg kg^−1^)	211 ± 9 a	211 ± 25 a	235 ± 9 ab	244 ± 32 ab	243 ± 27 ab	300 ± 22 b	459 ± 40 c	274 ± 15 ab	288 ± 22 b
W. soluble Na (mg L^−1^)	81.2 ± 5.1 c	9.9 ± 2.2 a	10.1 ± 2.4 a	12.9 ± 2.6 a	10.9 ± 1.9 a	43.5 ± 3.3 bc	32.1 ± 3.8 b	37.8 ± 2.4 b	48.1 ± 5.2 c
W. soluble K (mg L^−1^)	20 ± 2.6 b	12.8 ± 2.9 ab	8.5 ± 1.6 a	13.6 ± 2.9 ab	9.3 ± 1.9 a	24.6 ± 3.8 c	11.6 ± 1.9 a	11.7 ± 2.2 a	14.2 ± 3.4 ab
W. soluble Ca (mg L^−1^)	52.2 ± 3.5 c	18 ± 2.6 ab	13.2 ± 2.7 a	29.1 ± 3.2 b	20.5 ± 1.9 ab	59.9 ± 12.7 c	23.4 ± 2.7 ab	21.6 ± 2.1 ab	40.1 ± 5 bc
W. soluble Mg (mg L^−1^)	10.4 ± 1.8 b	3.8 ± 1 a	3.1 ± 1 a	5.5 ± 0.9 a	3.4 ± 1.1 a	16.8 ± 2.6 c	4.2 ± 0.7 a	4.4 ± 0.6 a	10.9 ± 1.5 b
W. soluble Cl (mg L^−1^)	144 ± 11.9 c	41.9 ± 3.7 b	11.9 ± 1.4 a	17.4 ± 3.3 a	12.4 ± 1.8 a	41.5 ± 3.8 b	34.4 ± 3.3 b	9.8 ± 1.4 a	33.5 ± 3 b
W. soluble SO_4_ (mg L^−1^)	61.1 ± 2.5 c	43.5 ± 3.3 c	8.6 ± 1.8 ab	15.7 ± 2.8 b	10.3 ± 2 ab	66 ± 3.4 c	37.6 ± 5.5 bc	6.6 ± 1.2 a	91.8 ± 4 c
W. soluble NO_3_ (mg L^−1^)	86.6 ± 6.9 c	18.6 ± 4.6 b	11.8 ± 1.9 ab	55.4 ± 5.2 c	16 ± 3.7 b	4.1 ± 0.6 a	15.7 ± 2 b	7 ± 1.3 ab	2.6 ± 0.7 a

EC: electrical conductivity, CEC: cation exchange capacity; W.: water; same letter means no significant differences (*p* < 0.05); Rhiz.: rhizospheric.

## Data Availability

The dataset is available upon request from the authors. The data are not publicly available due to confidentiality because the experimental work was carried out in private commercial farm.
